# Dietary Fatty Acids in Postprandial Triglyceride-Rich Lipoproteins Modulate Human Monocyte-Derived Dendritic Cell Maturation and Activation

**DOI:** 10.3390/nu12103139

**Published:** 2020-10-14

**Authors:** Carlos Vazquez-Madrigal, Soledad Lopez, Elena Grao-Cruces, Maria C. Millan-Linares, Noelia M. Rodriguez-Martin, Maria E. Martin, Gonzalo Alba, Consuelo Santa-Maria, Beatriz Bermudez, Sergio Montserrat-de la Paz

**Affiliations:** 1 Department of Medical Biochemistry, Molecular Biology and Immunology, School of Medicine, Universidad de Sevilla, 41009 Seville, Spain; vazquezmadrigal@gmail.com (C.V.-M.); slopez9@us.es (S.L.); egrao@us.es (E.G.-C.); galbaj@us.es (G.A.); 2 Department of Food & Health, Instituto de la Grasa, CSIC, 41013 Seville, Spain; mcmillan@ig.csic.es (M.C.M.-L.); noe91rm@gmail.com (N.M.R.-M.); 3 Department of Cell Biology, Faculty of Biology, Universidad de Sevilla, 41012 Seville, Spain; mariamartin@us.es (M.E.M.); bbermudez@us.es (B.B.); 4 Department of Biochemistry and Molecular Biology, School of Pharmacy, Universidad de Sevilla, 41012 Seville, Spain; csm@us.es

**Keywords:** fatty acids, postprandial state, chylomicron, olive oil, dendritic cells, myeloid lineage, triglyceride-rich lipoprotein

## Abstract

Dietary fatty acids have been demonstrated to modulate systemic inflammation and induce the postprandial inflammatory response of circulating immune cells. We hypothesized that postprandial triglyceride-rich lipoproteins (TRLs) may have acute effects on immunometabolic homeostasis by modulating dendritic cells (DCs), sentinels of the immunity that link innate and adaptive immune systems. In healthy volunteers, saturated fatty acid (SFA)-enriched meal raised serum levels of granulocyte/macrophage colony-stimulating factor GM-CSF (SFAs > monounsaturated fatty acids (MUFAs) = polyunsaturated fatty acids (PUFAs)) in the postprandial period. Autologous TRL-SFAs upregulated the gene expression of DC maturation (*CD123* and *CCR7*) and DC pro-inflammatory activation (*CD80* and *CD86*) genes while downregulating tolerogenic genes (*PD-L1* and *PD-L2*) in human monocyte-derived DCs (moDCs). These effects were reversed with oleic acid-enriched TRLs. Moreover, postprandial SFAs raised IL-12p70 levels, while TRL-MUFAs and TRL-PUFAs increased IL-10 levels in serum of healthy volunteers and in the medium of TRL-treated moDCs. In conclusion, postprandial TRLs are metabolic entities with DC-related tolerogenic activity, and this function is linked to the type of dietary fat in the meal. This study shows that the intake of meals enriched in MUFAs from olive oil, when compared with meals enriched in SFAs, prevents the postprandial production and priming of circulating pro-inflammatory DCs, and promotes tolerogenic response in healthy subjects. However, functional assays with moDCs generated in the presence of different fatty acids and T cells could increase the knowledge of postprandial TRLs’ effects on DC differentiation and function.

## 1. Introduction

The emerging research topic called immunometabolism investigates mutual interactions between the immune system and the metabolism [1]. Chronic low-grade inflammation and disturbances in immune cell population participate in metabolic disorders such as non-alcoholic fatty liver disease, type 2 diabetes mellitus, atherosclerosis, and metabolic syndrome [2]. Contrariwise, the dysfunctional remodeling of intracellular metabolic pathways plays a critical role for the functions of immune cells [3].

Dendritic cells (DCs) are the major antigen-presenting cells, which link the innate and adaptive immunity, maintaining tolerance to self-antigens. The human-derived DC family are typically classified into two phenotypically and functionally subsets, plasmacytoid DCs (pDCs) and myeloid DCs (mDCs) [4]. In bloodstream, in vitro homologous monocyte-derived DCs (moDCs), require the influence of granulocyte/macrophage colony-stimulating factor (GM-CSF) and interleukin-4 (IL-4) for differentiation [5]. Upon differentiation, moDCs undergo an activation process that, depending on their membrane receptors and secreted cytokines, means that moDCs may have either a pro-inflammatory or tolerogenic function. Pro-inflammatory activation is with CD80 and CD86 surface marker expression and IL-12p70 cytokine secretion, whereas PD-L1 and PD-L2 surface marker expression and IL-10 cytokine secretion are considered to prompt tolerogenic or anti-inflammatory activation of moDCs [6]. Any microenvironmental disturbance produces dysfunctional mDCs that may initiate inflammatory or autoimmune diseases. However, all the causes of mDC dysfunction have not been completely unraveled. One of the recognized causes is lipid accumulation, specifically triglycerides (TGs), in DCs from patients with cancer and autoimmune diseases, which may disturb DC function [7,8].

Owing to their hydrophobic nature, dietary fatty acids are transported in the form of postprandial TG-rich lipoproteins (TRLs, mainly chylomicrons) [9]. In healthy subjects, serum TG levels reach a peak over 1-3 h after eating a fatty meal, resulting in postprandial TRL accumulation in the bloodstream [10]. In previous studies, our research team has demonstrated the superiority of dietary oleic acid (i.e., monounsaturated fatty acid, MUFA) over palmitic and stearic acid (i.e., saturated fatty acids, SFAs) in buffering silent alterations postprandially [11,12,13]. Postprandial disturbances have been markedly interconnected with oxidative and inflammatory process linked to the differentiation and activation of circulating myeloid cells [14]. The binding of postprandial TRLs with their receptor, the ApoB48 receptor (ApoB48R), modulates TG accumulation in myeloid lineage [13,15], proposing that activation of immune cells may be the result of TRL uptake via ApoB48R. However, it is scarcely known whether dietary fatty acids in postprandial TRLs play a role in lipid accumulation in moDCs.

In this study, we assessed the potential role of TRLs rich in MUFAs (olive oil) without or with omega-3 long-chain polyunsaturated fatty acids (PUFAs)—olive oil + eicosapentaenoic acid (EPA) and docosahexaenoic acid (DHA)—compared to TRLs rich in SFAs (cow’s milk cream) in regulating moDC differentiation and whether dietary fatty acids are implicated in this process.

## 2. Materials and Methods

This study was conducted according to good clinical practice guidelines and in line with the principles outlined in the Helsinki Declaration of the World Medical Association. Ethics approval was obtained from the Human Clinical Research and Ethics Committee of the University Hospital Virgen Macarena (PI00082017) and all subjects gave written, informed consent.

### 2.1. Human Postprandial Study and TRL Isolation

Six volunteers, aged 25 to 35 years, non-smokers, with no medical history of disease known, nor any abnormality of hematological or biochemical parameters, were recruited in Clinical Biochemistry Unit at the University Hospital Virgen Macarena (UHVM, Seville). After an overnight fasting period of 12 h, all of them were given, over three different occasions, an oral fat emulsion containing cow’s milk cream (meal rich in SFAs), refined olive oil (meal rich in MUFAs) or refined olive oil plus a dose of omega-3 long-chain PUFAs, which consisted of 920 mg of EPA and 760 mg of DHA (a meal rich in PUFAs). They also consumed the same test meal without fat as a control meal. Oral fat emulsions were prepared according to the method described by our Patent WO/2014/191597. They consisted of water, sucrose, fat (50 g/m^2^ body surface area), emulsifier, and flavoring. At fasting (0 min) and after the ingestion of the meals within 10 min, blood samples were collected each hour into K3EDTA-containing Vacutainer tubes (Becton Dickinson, NJ, USA) over 6 h. Postprandial TRLs were isolated, pooled, and dialyzed against cold phosphate-buffered saline (PBS) [16]. TRLs were then immediately stored at −80 °C. Lipid oxidizability of postprandial TRL was checked (Thiobarbituric acid reactive substances level) during isolation and storage, but oxidation of lipids was not detected. TRLs were tested for lipopolysaccharide (LPS) contamination using the Pierce LAL Chromogenic Endotoxin Quantification kit (Thermo Scientific, Madrid, Spain). LPS contamination was always <0.2 EU/mL. TG concentration in postprandial TRLs was determined by colorimetric assay kit TG GPO-POD (Bioscience Medical, Madrid, Spain).

### 2.2. Fat and TRL Fatty Acid Composition

The fatty acid composition of cow’s milk cream, refined olive oil, and refined olive oil plus omega-3 long-chain PUFAs was determined, in triplicate from the same lot, by the method described in EEC/796/2002 [17] using a gas chromatography system (HP-5890, Hewlett-Packard, Waldbronn, Germany) equipped with flame ionization detector and a SP-2380 capillary column (Supelco, 30 m × 0.32 mm) packed with cyanopropylsiloxane (0.25 µm) (Supplementary Materials Table S1). The initial column temperature was 165 °C, which was held for 10 min, then programmed from 165 °C to 200 °C at 1.5 °C/min. Injector and detector temperature were 250 °C, with the carrier gas H_2_.

For fatty acid composition in postprandial TRLs (named TRL-SFAs from cow’s milk cream, TRL-MUFAs from refined olive oil, and TRL-PUFAs from refined olive oil plus omega-3 long-chain PUFAs), aliquots of 100 µL were lyophilized [18]. A solution composed of methanol: toluene: dimethoxypropane: sulphuric acid (16.5:5:1:1) and heptane was added on the lyophilized residue. After shaking and incubating the mixture at 80 °C for 1 h, the upper phase was transferred to another vial and dried with a stream of N_2_ gas. The resulting extract was dissolved in heptane and the FA methyl esters were analyzed into a gas chromatography system as described above (Table 1).

### 2.3. Monocyte Isolation

The same six volunteers who took part as donors of postprandial TRLs participated as donors of monocytes. After an overnight fasting period of 12 h, peripheral blood samples were drawn from a large antecubital vein and collected into K_3_EDTA-containing tubes (BD). Peripheral blood mononuclear cells (MNCs) were isolated by centrifugation over a Ficoll–Histopaque (Sigma, Madrid, Spain) gradient [19]. Monocytes were isolated from peripheral blood MNCs using anti-CD14 microbeads and LS columns on a midiMACS system (MiltenyiBiotec, Madrid, Spain). Monocyte (CD14^+^) purity was routinely >90% by flow cytometry analysis (FACSCanto II flow cytometer and FACSDiva software, BD) and cell viability >95% by trypan blue exclusion (Sigma). The monocytes were seeded in 24-well culture plates at a density of 1 × 10^6^ cells/mL and cultured in ultra-low attachment flasks in RPMI 1640 medium supplemented with L-glutamine, penicillin, streptomycin, and 10% heat-inactivated fetal bovine serum (complete culture medium).

### 2.4. MonocyteDerived Dendritic Cell Maturation and Activation

Monocytes were seeded in 24-well plates (1 × 10^6^ cells/well) and induced to differentiate for 6 days in the presence of human recombinant GM-CSF (50 ng/mL) and IL-4 (20 ng/mL) to obtain moDCs. Degree of differentiation of the resulting population was determined for CD123 antigen using anti-human CD123 monoclonal antibody (Miltenyi Biotec) by flow cytometry analysis (more than 95% of cells were positive for CD123) [20,21,22]. Complete culture medium was replaced every 2 days with fresh medium and the cytokines. To study the effect of TRLs on moDC differentiation, monocytes were treated for 6 days with TRL-SFAs, TRL-MUFAs, or TRL-PUFAs at 100 µg TG/mL in presence of GM-CSF and IL-4.

### 2.5. Monocyte-Derived Dendritic Cell Viability

For cell viability, monocytes were seeded in 96-well plates (1 × 10^5^ cells/well) and differentiated into moDCs as indicated above. At days 1, 2, 4, and 6, 3-(4,5-dimethylthiazol-2-yl)-2,5-diphenyltetrazolium bromide (MTT) solution (Sigma) was added to cells for 2 h until a purple precipitate was visible. MTT–formazan crystals were then solubilized with dimethyl sulfoxide (DMSO) (Sigma) and measured with a microplate reader at 570 nm corrected to 650 nm. Cell survival was expressed as the percentage of absorbance compared with that of the control, non-treated cells.

### 2.6. Triglyceride Quantification

Cellular lipids were extracted using hexane/isopropanol (3:2, *v*/*v*). The supernatant was obtained after centrifugation at 500× *g* for 5 min. The TG content was measured using the assay kits GPO/PAP (Axiom Diagnostics, Burstadt and Worms, Germany). To determine the protein content, cells were sonicated in radioimmunoprecipitation assay (RIPA) buffer, and the lysate was measured using the Bradford protein assay (Bio-Rad Laboratories, Madrid, Spain).

### 2.7. RNA Isolation and RT-qPCR

Total RNA was extracted by using Trisure Reagent (Bioline). RNA quality was assessed by A_260_/A_280_ ratio in a NanoDrop ND-1000 Spectrophotometer (Thermo Scientific). Briefly, RNA (1 µg) was subjected to reverse transcription (iScript, Bio-Rad, Madrid, Spain). An amount of 10 ng of the resulting cDNA was used as a template for real-time PCR amplifications. The mRNA levels for specific genes were determined in a CFX96 system (Bio-Rad). For each PCR reaction, cDNA template was added to Brilliant SYBR green QPCR Supermix (Bio-Rad) containing the primer pairs for either gene or for glyceraldehyde 3-phosphate dehydrogenase (*GAPDH*) and hypoxanthine phosphoribosyltransferase (*HPRT*) as housekeeping genes (Supplementary Materials Table S2). All amplification reactions were performed in triplicate and average threshold cycle (Ct) numbers of the triplicates were used to calculate the relative mRNA expression of candidate genes. The magnitude of change of mRNA expression for candidate genes was calculated by using the standard 2^−(ΔΔCt)^ method. All data were normalized to endogenous reference (*GAPDH* and *HPRT*) gene content and expressed as relative fold-change of control.

### 2.8. Cytokine Quantification

The cytokines GM-CSF, IL-12p70, and IL-10 were determined by enzyme-linked immunosorbent assay (ELISA), following the indications of the manufacturer (Diaclone, Besançon, France). Cytokine concentration was expressed in *p*g/mL, as calculated from the calibration curves from serial dilution of human recombinant standards in each assay.

### 2.9. Statistical Analysis

All values are expressed as arithmetic means ± standard deviations (SD). Data were evaluated with Graph Pad Prism Version 5.01 software (San Diego, CA, USA). The statistical significance of any difference in each parameter among the groups was evaluated by one-way analysis of variance following Tukey’s multiple comparisons test as a post hoc test. The Pearson *r* value was used to analyze the statistical significance of correlation test. *p* values less than 0.05 were considered statistically significant.

## 3. Results

### 3.1. Dietary Saturated Fatty Acids Acutely Increase Serum GM-CSF Levels in the Postprandial State of Healthy Volunteers

**Figure 1:**
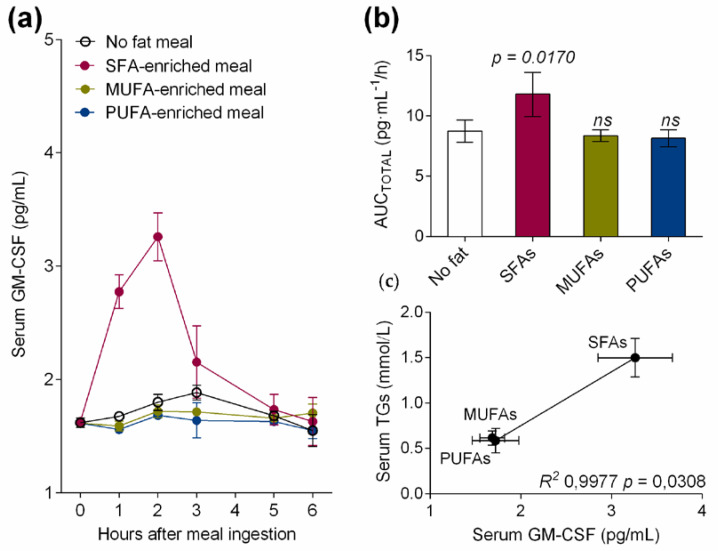
(**a**) Serum granulocyte/macrophage colony-stimulating factor (GM-CSF) levels at fasting and at the postprandial period after the administration of a control meal (with no fat) or high-fat meals enriched in saturated fatty acids (SFAs), monounsaturated fatty acids (MUFAs) or MUFAs + omega-3 long-chain polyunsaturated fatty acids (PUFAs) in healthy subjects. (**b**) Area under the curve of serum GM-CSF during postprandial period in response to test meals. (**c**) Correlation between postprandial serum GM-CSF levels and serum triglycerides (TGs) in healthy subjects after administration of test meals. Values are presented as means ± SD (*n* = 6).

Serum from healthy volunteers was collected at fasting (TG concentration 0.42 ± 0.1 mmol/L) and at the postprandial hypertriglyceridemic peak (1–3 h) after SFA-enriched (TG concentration 1.54 ± 0.4 mmol/L), MUFA-enriched (TG concentration 0.65 ± 0.2 mmol/L), or PUFA-enriched (TG concentration 0.63 ± 0.2 mmol/L) meal ingestion. As depicted in Figure 1a, only the meal rich in SFAs postprandially increased serum GM-CSF levels when compared to the other fatty acid-enriched and no-fat meals. Thus, the total area under the curve (AUC_TOTAL_) values for serum GM-CSF were significantly higher (*p* = 0.0170) only after the ingestion of the high-fat meal enriched in SFAs in healthy volunteers (Figure 1b). Interestingly, serum GM-CSF levels, which is a DC maturation factor, were acutely increased by the SFA-enriched meal at the postprandial hypertriglyceridemic peak, suggesting that dietary fatty acids present in TRLs modulate the DC maturation process. In addition, serum TG correlated with the levels of serum GM-CSF in healthy volunteers (*R*^2^ 0.9977, *p* = 0.0308, Figure 1c).

### 3.2. Triglyceride Rich-Lipoproteins Modulate Maturation and Activation Markers in Human Monocyte-Derived Dendritic Cells

**Figure 2:**
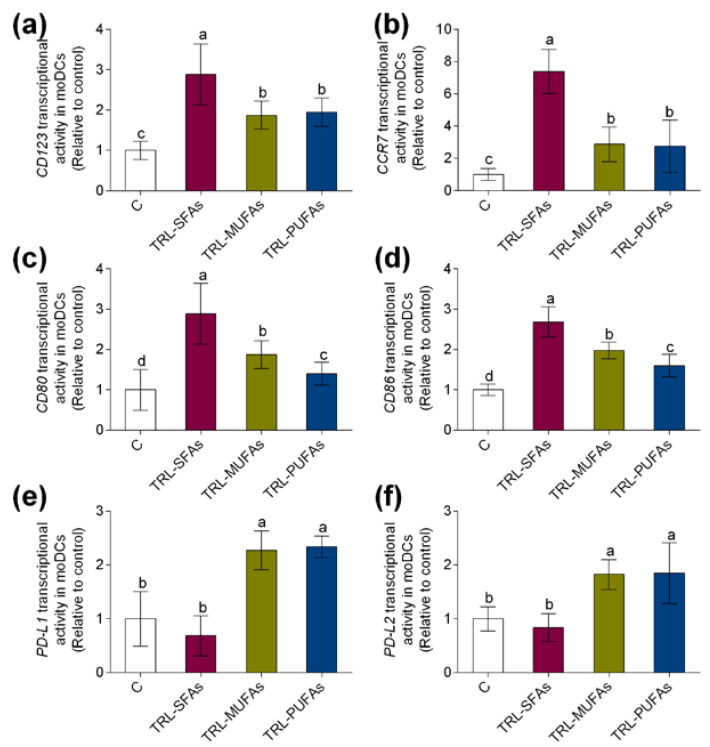
In vitro expression of dendritic cell (DC) gene markers in monocyte-derived DCs (moDCs) after stimulation with TRL-SFAs, TRL-MUFAs and TRL-PUFAs (TRL, triglyceride-rich lipoprotein) at 100 µg of TGs/mL for 6 days and in presence of GM-CSF and interleukin-4 (IL-4). DC maturation markers: (**a**) CD123 and (**b**) CCR7. DC pro-inflammatory activation markers: (**c**) CD80 and (**d**) CD86. DC tolerogenic activation markers: (**e**) PD-L1 and (**f**) PD-L2. Control means non-treated cells in the presence of GM-CSF and IL-4. Values are presented as means ± SD (*n* = 6) and those marked with different letters are significantly different (*p* < 0.05).

In gaining deeper insight into the role of postprandial TRLs in DC maturation and activation, marker gene expressions during human monocyte differentiation into moDCs were studied. Postprandial TRLs at 100 µg TGs/mL added to the differentiation medium that contained GM-CSF and IL-4 for 6 days did not induce cytotoxicity (data not shown). In previous reports, postprandial TRLs were not cytotoxic to human monocyte-derived osteoclasts [9] and macrophages [15] at similar concentrations. As shown in Figure 2, both transcriptional activity of *CD123* (Figure 2a) and *CCR7* (Figure 2b), key regulators of DC maturation, were upregulated by TRL-MUFAs and TRL-PUFAs but more markedly by TRL-SFAs, suggesting that postprandial fatty acids present in TRLs, in a saturation degree-dependent manner, modulate the DC maturation process. To further explore the possible pro-inflammatory or tolerogenic effects of postprandial TRLs in human moDCs, gene expression of CD80 (Figure 2c) and CD86 (Figure 2d) pro-inflammatory activation markers and PD-L1 (Figure 2e) and PD-L2 (Figure 2f) tolerogenic activation markers were analyzed. Postprandial TRLs affected the activation status of moDCs. Interestingly, TRL-SFAs upregulated the postprandial-TRL-induced transcriptional activity of CD80 and CD86 pro-inflammatory gene markers in moDCs. In contrast, TRL-MUFAs and TRL-PUFAs induced an upregulation in PD-L1 and PD-L2 gene expression, whereas TRL-SFAs did not induce any changes compared to control gene expression.

### 3.3. Fatty Acid-Enriched Meals Modulate Serum IL-12p70 and IL-10 Postprandial Secretion and Triglyceride-Rich Lipoproteins Regulate Cytokine Levels and Gene Expression in Human Monocyte-Derived Dendritic Cells

**Figure 3:**
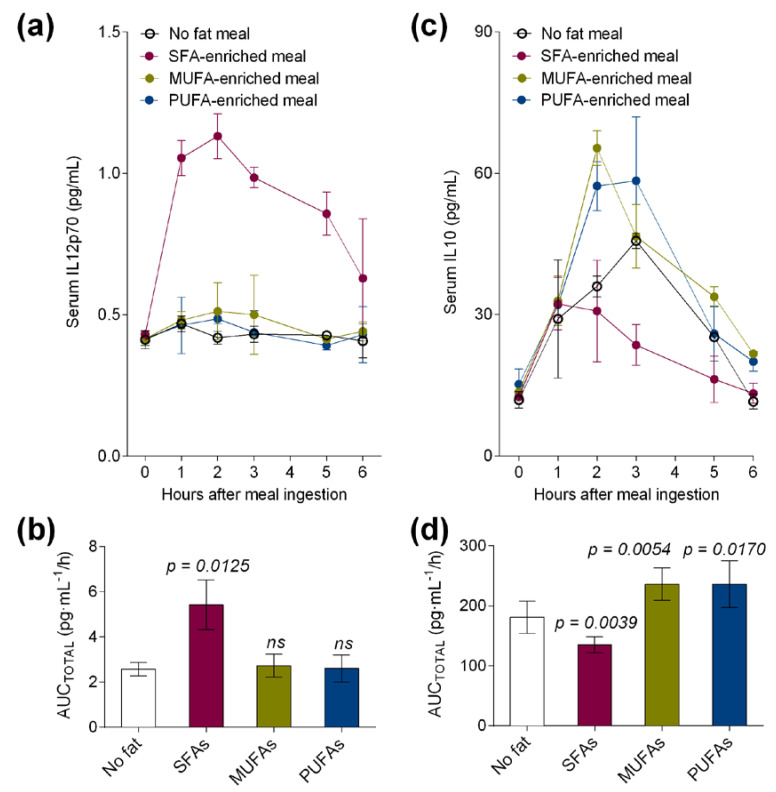
(**a**) Serum and (**b**) area under the curve of pro-inflammatory IL12p70 levels and (**c**) serum and (**d**) area under the curve of tolerogenic IL10 levels at fasting and at postprandial period after the administration of a control meal (with no fat) or high-fat meals enriched in SFAs, MUFAs, or MUFAs + omega-3 LCPUFAs (PUFAs) in healthy subjects. Values are presented as means ± SD (*n* = 6) and those marked with different letters are significantly different (*p* < 0.05). AUC: area under the curve.

We also investigated the secretion levels of pro-inflammatory IL-12p70 and tolerogenic IL-10 in postprandial serum of healthy volunteers. After meal ingestion, postprandial serum levels of IL-12p70 (Figure 3a) were increased by the SFA meal but not by the MUFA or PUFA meal when compared to the control meal with no fat. Remarkably, IL-12p70 AUC_TOTAL_ was particularly increased by the SFA meal (*p* = 0.0125, Figure 3b) over postprandial 6 h. In contrast, IL-10 levels were postprandially lower after the SFA meal (Figure 3c) and higher after the MUFA and PUFA meal ingestion. Thus, AUC_TOTAL_ values for serum IL-10 were significantly lower (*p* = 0.0039) only after the ingestion of the high-fat meal enriched in SFAs and higher after the ingestion of the MUFA- (*p* = 0.0054) and PUFA- (*p* = 0.0170) enriched meals in healthy volunteers when compared to the control meal with no fat (Figure 3d).

**Figure 4:**
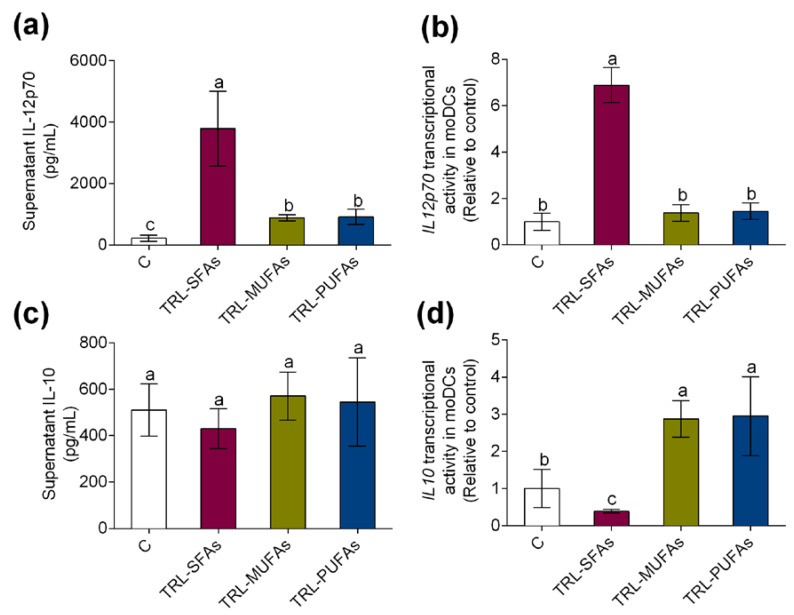
In vitro expression and secretion of proinflammatory and tolerogenic cytokines in moDCs after stimulation with TRL-SFAs, TRL-MUFAs, and TRL-PUFAs at 100 µg of TGs/mL for 6 days and in presence of GM-CSF and IL-4. (**a**) IL-12p70 secretion and (**b**) mRNA expression. (**c**) IL-10 secretion and (**d**) mRNA expression. Values are presented as means ± SD (*n* = 6) and those marked with different letters are significantly different (*p* < 0.05).

In line with these results, in vitro experiments showed that TRL-SFAs promoted the secretion and transcriptional activity of *IL12p70* (Figure 4a and Figure 4b, respectively). On the other hand, no changes were observed on the IL-10 secretion in moDCs (Figure 4c); however, TRL-SFAs downregulated *IL10* transcriptional activity in moDCs (Figure 4d). Contrary to SFAs, TRL-MUFAs and TRL-PUFAs upregulated tolerogenic *IL10* transcriptional activity in moDCs.

### 3.4. Triglyceride-Rich Lipoproteins Induce Lipid Accumulation and ApoB48R Transcriptional Activity in Monocyte-Derived Dendritic Cells in a Fatty Acid-Dependent Manner

**Figure 5:**
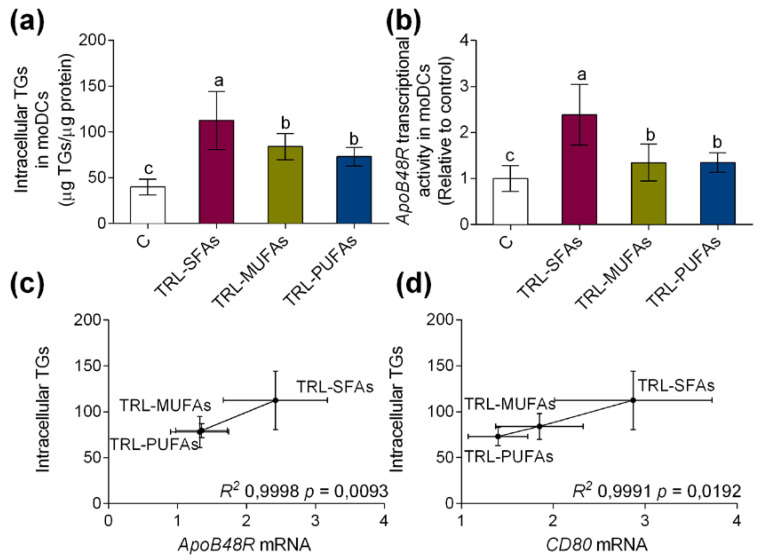
(**a**) Intracellular TGs accumulation and (**b**) In vitro *apoB48R* mRNA induced in moDCs after stimulation with TRL-SFAs, TRL-MUFAs and TRL-PUFAs at 100 µg of TGs/mL for 6 days and in presence of GM-CSF and IL-4. (**c**) Correlation between *apoB48R* mRNA and (**d**) *CD80* mRNA (DC activation marker) with intracellular TGs in moDCs. Control means non-treated cells in presence of GM-CSF and IL-4. Values are presented as means ± SD (*n* = 6) and those marked with different letters are significantly different (*p* < 0.05). TGs: triglycerides.

Finally, we want to establish whether lipid accumulation would function to induce moDC activation. TRL-SFAs induced higher increase in TG accumulation (Figure 5a) and *ApoB48R* transcriptional activity (Figure 5b) compared those induced by TRL-MUFAs and TRL-PUFAs in moDCs. In addition, intracellular TG correlated with the expression of the ApoB48R (*R*^2^ 0.9998, *p* = 0.0093, Figure 5c) and *CD80 (R*^2^ 0.9991, *p* = 0.0192, Figure 5d) activation marker, suggesting that dietary FAs present in TRLS, in a saturation degree-dependent manner, intervene in the activation process of moDCs.

## 4. Discussion

The literature often suggests that lifestyle and traditional dietary habits unique to the Mediterranean region play a role in the prevention of oxidative- and inflammatory-related pathologies, such as cardiometabolic diseases and cancer [23]. Olive oil, the main dietary fat in the Mediterranean diet, due to its content of oleic acid (MUFA) and minor constituents, modulate different processes linked to chronic low-grade inflammation [24]. This view is in contrast to diets rich in SFAs, such as the “meat-based” or “Westernized” diets, which are inductive of inflammatory states [25]. One of the key processes of inflammation is the maturation and activation of circulating myeloid cells. These leukocytes are the first immune cells that respond quickly to injury and their activation, if permanent or chronic, may cause the increase of the inflammatory response, the perpetuation of the inflammatory state and the development of obesity or autoimmune disease [26,27]. DCs are professional antigen-presenting cells within the immune system, that are uniquely capable of priming naïve T cells, and once activated they have a pivotal ability to induce primary innate and adaptive immune response [28]. However, little is known about the effect of dietary fatty acids on human moDC [29]. The experimental use of primary human DCs is limited by their rarity in peripheral blood (less than 1% of MNCs), so to avoid this, in vitro moDCs are generally selected as a pragmatic model [30].

The postprandial period, the state that comprises and follows a meal, has an important, yet underrated, role in the onset of several pathologies. After fatty meal intake, dietary fatty acids are largely integrated into nascent TRLs, which are liberated from the small intestine into the bloodstream. It has been previously reported that dietary fatty acids have divergent postprandial effects on chronic disease-related events [31], suggesting that acute outcomes in response to dietary SFA-, MUFA- or PUFA-adjustment may be helpful to lightly attenuate, even for preventing, diet-related chronic diseases [10]. It is essential to mention that the postprandial period is defined by a large number of metabolic transformations that comprise the raise of circulating TRLs. In response to fatty meal intake, earlier human studies have demonstrated the association of activated myeloid cells with postprandial hyperlipidemia [32,33]. Notwithstanding the relevance, studies during the postprandial period on interaction between human postprandial TRLs and moDCs are still unknown. Our results show for the first time that, after SFA-enriched meal intake, postprandial hypertriglyceridemia is associated with an increase of serum GM-CSF in healthy subjects. This effect was regulated by the main fatty acids in dietary fats, being significantly raised after the ingestion of an SFA-enriched meal when compared to the ingestion of MUFA-enriched meals. In line with these results, McFarlin et al. demonstrated that high-calorie meals significant increased postprandial GM-CSF and G-CSF levels in humans [34].

In gaining deeper insight into the role of dietary fatty acids on differentiation of moDCs, we obtained fresh monocytes from the fasting blood samples of healthy volunteers. Then, cells were differentiated into moDCs (GM-CSF + IL4 treatment for 6 days) in the absence or the presence of TRL-SFAs, TRL-MUFAs, and TRL-PUFAs isolated from postprandial serum samples of the same volunteers. In these experimental setting of autologous interaction, we observed an upregulation of DC maturation (CD123 and CCR7) and pro-inflammatory activation (CD80 and CD86) markers, and a downregulation of tolerogenic activation (PD-L1 and PD-L2) markers in human moDCs in response to postprandial TRL-SFAs. These effects support the notion that dietary saturated fats promote pro-inflammatory functions in mature DCs through metabolic pathways involving lipoproteins. In line with these results, Nicholas et al. demonstrated that palmitic acid-stimulated moDCs upregulated the expression of CD83 and CD86 [35]. Additionally, palmitic acid induced TLR4-dependent secretion of IL-1β, generated reactive oxygen species, and activated the NFκB canonical pathway in moDCs [35]. Importantly, our study showed a significant attenuation of incremental DC maturation and activation following the treatment with TRL-MUFAs and TRL-PUFAs, suggesting that the replacement of dietary SFAs by MUFAs (in combination or not with omega-3 long-chain PUFAs) could be helpful to prevent excessive DC-associated with postprandial events. Our new data extend the previous in vitro studies with PUFAs, and emphasize their acute benefits on TRLs to a healthy population. In line with this notion, moDCs stimulated with DHA and EPA show a reduction in the expression of CD80 and CD86 and in the secretion of IL-12p70 [36]. To our best knowledge, for the first time, the current study demonstrates that oleic acid from olive oil decreases, even abrogates, the gene expression of DC maturation and activation gene markers and the pro-inflammatory cytokine release. However, functional assays with moDCs generated in the presence of different fatty acids and T cells could increase the knowledge of postprandial TRLs’ effects on DC differentiation and function.

Finally, in line with previous data in human neutrophils [37], monocytes [15,38], and murine microglia [13], our study have showed that postprandial TRLs induced DC activation through ApoB48R upregulation in a FA-dependent manner. Dietary oleic acid, EPA, and DHA attenuated ApoB48R gene expression while triggering a depletion in intracellular TG accumulation compared to palmitic acid.

## 5. Conclusions

In conclusion, these findings suggest that dietary fatty acids play a relevant and interrelated role in protecting against DC postprandial differentiation. Our results open new opportunities for developing novel nutritional strategies with olive oil as the principal dietary source of MUFAs, notably oleic acid, to prevent development and progression of inflammatory- and autoimmune-related diseases.

## Figures and Tables

**Table 1:** Fatty acid composition of postprandial TRLs.

Fatty Acid	TRL-SFAs	TRL-MUFAs	TRL-PUFAs
	g/100 g of Fatty Acid		
4:0, butyric	0.26 ± 0.03	-	-
6:0, caproic	0.19 ± 0.02	-	-
8:0, caprylic	0.38 ± 0.14	-	-
10:0, capric	1.62 ± 0.52	-	-
12:0, lauric	3.52 ± 1.01	-	-
14:0, myristic	8.76 ± 1.63	-	-
16:0, palmitic	38.10 ± 1.87	11.2 ± 1.52	11.98 ± 1.21
16:1(n-7), palmitoleic	1.03 ± 0.10	0.79 ± 0.21	1.42 ± 0.61
18:0, stearic	18.8 ± 1.32	5.71 ± 0.73	5.54 ± 0.78
18:1(n-9), oleic	20.7 ± 1.76	67.2 ± 2.97	61.3 ± 3,87
18:2(n-6), linoleic	4.04 ± 0.98	8.95 ± 1.32	9,06 ± 1.03
18:3(n-3), α-linolenic	1.96 ± 0.57	3.29 ± 0.74	3.03 ± 0.98
20:4(n-6), arachidonic	0.49 ± 0.09	1.18 ± 0.37	1.79 ± 0.42
20:5(n-3), eicosapentaenoic	-	0.78 ± 0.19	2.82 ± 0.29
22:6(n-3), docosahexaenoic	-	0.70 ± 0.28	2.63 ± 0.14
Others	0.51 ± 0.13	0.34 ± 0.07	0.56 ± 0.07

Data are expressed as mean ± SD, *n* = 18. TRL: triglyceride-rich lipoprotein; SFAs:saturated fatty acids; MUFAs: monounsaturated fatty acids; PUFAs: polyunsaturated fatty acids.

## Supplementary Materials

The following are available online at https://www.mdpi.com/2072-6643/12/10/3139/s1, Table S1: Fatty acid composition of dietary fats, Table S2: Detailed information about primers’ sequences used in this study.

## Abbreviations

DC	dendritic cell
GM-CSF	granulocyte/macrophage colony stimulating factor
IL	interleukin
mDC	myeloid dendritic cell
moDC	monocyte-derived dendritic cell
MUFA	monounsaturated fatty acid
pDC,	plasmacytoid dendritic cell
SFA	saturated fatty acid
TG	triglyceride
TRL	triglyceride-rich lipoprotein
