# Supplementary material for: Dietary Fatty Acids in Postprandial Triglyceride-Rich Lipoproteins Modulate Human Monocyte-Derived Dendritic Cell Maturation and Activation
Source: Nutrients. 2020 Oct 14;12(10):3139. doi: 10.3390/nu12103139 (PMC7656296; doi:10.3390/nu12103139)
Supplement: Supplementary file 1 [file nutrients-12-03139-s001.docx]

**Supporting Information Table 1.** Fatty acid composition of dietary fats.

|  | **Cow's milk cream** | **Refined olive oil** | **Refined olive oil + omega-3 long-chain PUFAs** |
| --- | --- | --- | --- |
| **Fatty acid** | **g/100 g of fatty acid** | | |
| 4:0, butyric | 0.81 ± 0.10 | - | - |
| 6:0, caproic | 0.28 ± 0.03 | - | - |
| 8:0, caprylic | 0.63 ± 0.08 | - | - |
| 10:0, capric | 2.41 ± 0.11 | - | - |
| 12:0, lauric | 3.15 ± 0.38 | - | - |
| 14:0, myristic | 11.2 ± 0.81 | - | - |
| 16:0, palmitic | 35.9 ± 0.94 | 18.4 ± 1.36 | 20.1 ± 0.53 |
| 16:1(n-7), palmitoleic | 3.65 ± 0.42 | 1.11 ± 0.21 | 0.91 ± 0.11 |
| 18:0, stearic | 11.25 ± 0.65 | 4.98 ± 0.41 | 5.09 ± 0.54 |
| 18:1(n-9), oleic | 25.1 ± 0.83 | 63.2 ± 2.08 | 61.0 ± 1.07 |
| 18:2(n-6), linoleic | 4.26 ± 0.65 | 8.41 ± 0.75 | 7.99 ± 0.71 |
| 18:3(n-3), α-linolenic | 0.40 ± 0.02 | 1.31 ± 0.22 | 0.93 ± 0.08 |
| 20:5(n-3), eicosapentaenoic | - | - | 0.99 ± 0.11 |
| 22:6(n-3), docosahexaenoic | - | - | 0.82 ± 0.10 |
| Others | 0.95 ± 0.48 | 2.63 ± 1.12 | 2.24 ± 0.93 |

Data are expressed as mean ± SD, *n* = 3.

**Supporting Information Table 2.** Detailed information about primers’ sequences used in this study.

| **Target** | **GenBank accession Number** | **Direction** | **Sequence (5´🡪3´)** |
| --- | --- | --- | --- |
| *HPRT* | NM_000194 | Forward  Reverse | ACCCCACGAAGTGTTGGATA  AAGCAGATGGCCACAGAACT |
| *GAPDH* | NM_001289746 | Forward  Reverse | CACATGGCCTCCAAGGAGTAAG  CCAGCAGTGAGGGGTCTCTCT |
| *CD123* | NM_002183 | Forward  Reverse | AGGCGTCAACAGTACGAGTG  CCACCAGCTTGTCGTTTTGG |
| *CCR7* | NM_001301716.1 | Forward  Reverse | TGGAGGCCTTTATCACCATC  TGTAGGGCAGCTGGAAGACT |
| *CD80* | NM_005191.3 | Forward  Reverse | ATGCTGCCTGACCTACTGCT  GGTCAATTGCAAATGGAGGT |
| *CD86* | NM_175862.5 | Forward  Reverse | TAGGTCACAGCAGAAGCAGC  AATCAAAACTTGTGCGGCCC |
| *PD-L1* | NM_014143 | Forward  Reverse | CACGGTTCCCAAGGACCTAT  TGGAGGATGTGCCAGAGGTA |
| *PD-L2* | NM_025239 | Forward  Reverse | AGAGGGAAGTGAACAGTGCT  TAGGCTCCAGAGGTGAGTCC |
| *IL12p70* | NM_002187 | Forward  Reverse | TGTGATGGATGGGAACGCAA  GGGCTGTTAAGAAGCCACCT |
| *IL10* | [NM_000572](http://www.ncbi.nlm.nih.gov/entrez/viewer.fcgi?val=NM_000572) | Forward  Reverse | CTGACATCAAGGAGCACGTG  GGCTTTGTAGACACCCCTCT |
| *ApoB48R* | NM_018690.4 | Forward  Reverse | TCACACACAGCGTCACCAAA  TGTCCTTTCTCGTGCGGTTT |
